# Harnessing Bacteriophages for Sustainable Crop Protection in the Face of Climate Change

**DOI:** 10.1111/1751-7915.70108

**Published:** 2025-02-12

**Authors:** Robert Czajkowski, Amalia Roca, Miguel A. Matilla

**Affiliations:** ^1^ Laboratory of Biologically Active Compounds Intercollegiate Faculty of Biotechnology UG and MUG, University of Gdansk Gdansk Poland; ^2^ Department of Microbiology, Facultad de Farmacia Campus Universitario de Cartuja, Universidad de Granada Granada Spain; ^3^ Department of Biotechnology and Environmental Protection Estación Experimental del Zaidín, Consejo Superior de Investigaciones Científicas Granada Spain

## Abstract

Crop pathogens represent a major challenge to global food security, causing over 40% yield losses in key crops and annual economic impacts estimated at up to US$290 billion. Microbial‐based alternatives to synthetic agrochemicals offer sustainable solutions aligned with global initiatives like the European Union's Green Deal. Among these, bacteriophage (phage) therapy has gained attention for its specificity, effectiveness against plant pathogens and safety for crops. Here, we highlight recent research on phage therapy strategies and their potential utility in sustainable agriculture, showcasing its effectiveness in reducing phytopathogen densities, delaying plant disease onset, and enriching plant‐associated bacterial taxa with biocontrol potential. Phage cocktails improve biocontrol, mitigate resistance, and synergize with other biological and chemical agents. Emerging technologies like engineered phages also promise enhanced efficacy. Addressing challenges like phytopathogen resistance, field inconsistencies, and regulatory hurdles is crucial to integrating phage therapy into sustainable agriculture under climate stress.

## Graphical abstract

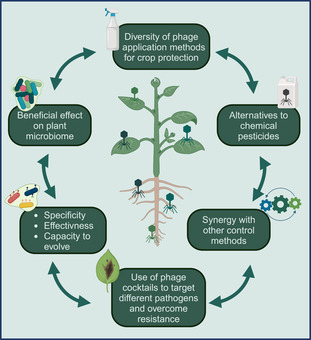

Crop pathogens are among the primary factors limiting agricultural production and have a significant impact on global food safety. Although precise estimates of the damage caused by these pathogens are challenging to determine, some studies indicate that they contribute to up to 41% of global losses in major crops (Savary et al. 2019). This translates to an annual economic impact estimated at approximately US$220‐290 billion (Holtappels et al. 2021; Singh et al. 2023). In this context, microorganism‐based methods offer promising and sustainable alternatives to antibiotics, chemical fertilisers and pesticides, which pose significant risks to public health and the environment (Carlos 2020, Holtappels et al. 2021; Singh et al. 2023; Toussaint et al. 2024; Warring et al. 2024). These microbial‐based solutions to synthetic agrochemicals include biostimulants, biofertilizers and biopesticides, as well as advances in microbiome engineering (Panda and Zhou 2023; Roca and Matilla 2023; Woo et al. 2023; Crowther et al. 2024; Roca et al. 2024; Tran et al. 2024). The development of these ecologically friendly alternatives is supported by policies such as the European Union's Green Deal, which aims to achieve a 50% reduction in the use of synthetic agrochemicals by 2030 (European Union: sustainable use of pesticides 2024). Beyond these microbial‐based approaches, bacteriophages (or phages—viruses that infect bacteria) were first used to treat phytopathogens in the early 1920s (Holtappels et al. 2021). Today, phage therapy is gaining increasing interest as a biocontrol strategy because of several advantages: (i) high specificity; (ii) ability to reduce target bacterial populations; (iii) effectiveness against multi‐drug resistant bacteria; (iv) capacity to co‐evolve with phage‐resistant bacteria, preserving long‐term effectiveness; (v) versatility in crop application methods (e.g., plant sprays, seed coatings, incorporation into soil treatment formulations); and (vi) the absence of known negative effects on plants and other eukaryotic organisms (Holtappels et al. 2021; Erdrich et al. 2024; Ke et al. 2024; Siyanbola et al. 2024; Toussaint et al. 2024; Warring et al. 2024). Rhizosphere‐associated phages significantly influence rhizosphere bacterial community structure and nutrient turnover (Pratama et al. 2020; Yang et al. 2023), with healthy plants exhibiting a higher abundance of phages targeting phytopathogens as compared to diseased plants (Yang et al. 2023). In fact, phage therapy has demonstrated efficiency in the biocontrol of globally relevant phytopathogens such as *Pseudomonas syringae* , *Xylella fastidiosa* , *Xanthomonas campestris* , *Xanthomonas oryzae* , *Pectobacterium carotovorum* , *Ralstonia solanacearum* , *Erwinia amylovora* and *Dickeya solani* (Wang et al. 2019; Holtappels et al. 2021; Yang et al. 2023; Ke et al. 2024; Siyanbola et al. 2024; Toussaint et al. 2024; Warring et al. 2024). Although still in its early stages of agricultural application, several phage‐based products are already commercially available (Table 1).

**TABLE 1:** Phage‐based products for agricultural use.

Phage product	Target phytopathogen	Certifications (regulatory approval)	Company (Country)
AgriPhage—Spot & Speck	*Xanthomonas* spp.; *Pseudomonas syringae* pv. *tomato*	USA/Canada (Environmental Protection Agency [EPA] registered)	Omnilytics Inc. (USA)
AgriPhage—Tomato Canker	*Clavibacter michiganensis* subsp. *michiganensis*		
AgriPhage—Fire Blight	*Erwinia amylovora*		
AgriPhage^®^—Citrus Canker	*Xanthomonas citri* pv. *citri*		
AgriPhage—Nut & Stone Fruit	*Xanthomonas arboricola* pv. *pruni*; *Xanthomonas arboricola* pv. *juglandis*; *Xanthomonas arboricola* pv. *corylina*; *Pseudomonas syringae* pv. *syringae*		
Erwiphage PLUS	*Erwinia amylovora*	Hungary (undefined)^a^	Enviroinvest Corp. (Hungary)
XylPhi‐PD	*Xylella fastidiosa*	USA (Environmental Protection Agency [EPA] registered)	A&P Inphatec (USA)
Biolyse technology	Development of phage cocktails for the treatment of crops, such as targeting soft‐rot bacteria (*Pectobacterium* spp.), as well as for post‐harvest applications.	Patented technology (undefined)	APS Biocontrol Ltd. (United Kingdom)
BPSRE (bacteriophage of Potato Soft Rot *Enterobacteriaceae*)	Potato soft rot *Enterobacteriaceae*	Europe (pending approval. Active substance ID. 1483)^b^	—
BAEA (bacteriophages against *Erwinia amylovora* )	*Erwinia amylovora*	Europe (pending approval. Active substance ID. 1514)^b^	—

^a^ Temporary authorization during spring 2020 for local sales in Hungary under strict regulations (Holtappels et al. 2021).

^b^ No phage‐based products are currently registered by the ‘European Food Safety Authority’ as plant protection products or biopesticides (see: https://ec.europa.eu/food/plant/pesticides/eu‐pesticides‐database/start/screen/active‐substances; Accessed on 30 December 2024).

The use of phage cocktails is regarded as a strategy to enhance biocontrol efficiency by simultaneously targeting different phytopathogens and reducing resistance development (Wang et al. 2019; Holtappels et al. 2021; Yang et al. 2023; Ke et al. 2024; Siyanbola et al. 2024; Warring et al. 2024). Additionally, phage treatments have the potential to influence microbial interactions within plant niches (Wang et al. 2019; Yang et al. 2023; Siyanbola et al. 2024), potentially enhancing the efficiency of biocontrol treatments. In this regard, two recent studies explored the effectiveness of individual phages and phage cocktails in suppressing disease caused by *R. solanacearum* (Franco Ortega et al. 2024; Wang et al. 2024), one of the world's top 10 bacterial phytopathogens (Mansfield et al. 2012). In one study, Franco Ortega et al. (2024) found that phage treatments, whether as individual phages or cocktails, delayed disease development in greenhouse experiments with tomato plants. This delay correlated with a reduction in *R. solanacearum* levels and with increased phage densities. Analysis of rhizosphere bacterial microbiomes after phage treatments revealed reduced bacterial diversity in phage‐treated plants compared to untreated plants, with specific bacterial genera significantly enriched in the rhizospheres of phage‐treated plants (Franco Ortega et al. 2024). To assess the functional relevance of these microbiome changes, the authors evaluated the antagonistic properties of the enriched taxa against *R. solanacearum*. It was found that rhizosphere isolates from genera such as *Pseudomonas*, *Rhodanobacter* and *Burkholderia* demonstrated in vitro antagonism against *R. solanacearum* . Notably, several *Burkholderia* isolates showed strong biocontrol properties *in planta* against *R. solanacearum* , supporting that microbiome changes contribute to the suppressiveness of rhizospheric bacterial communities. Partial least squares path modelling revealed that phage‐induced changes in the rhizosphere microbiota played an important role in plant disease incidence (Franco Ortega et al. 2024). In a complementary study, Wang et al. (2024) showed that repeated applications of a phage cocktail significantly enhanced biocontrol efficacy against *R. solanacearum* in both greenhouse and field experiments, with this efficacy correlating to reduced phytopathogen densities. Importantly, the authors found no evidence of phage resistance emerging during the treatments (Wang et al. 2024), consistent with earlier *in planta* studies (Meaden et al. 2015). Greenhouse trials revealed that phage treatments increased rhizosphere bacterial diversity and richness, with repeated applications amplifying these effects. In addition, the reduction of *R. solanacearum* correlated positively with phage proliferation in the rhizosphere and increased abundances of bacterial phyla such as Gemmatimonadota, Actinomycetota, Acidobacteriota and Myxococcota. Among these taxa, Actinomycetota has a high genetic potential to synthesise bioactive secondary metabolites (Gavriilidou et al. 2022; Blin et al. 2023). Greenhouse trials recovered several *Nocardioides* and *Streptomyces* isolates from the rhizosphere of healthy, phage‐treated plants, and these isolates showed inhibitory effects against *R. solanacearum* both in vitro and *in planta*. Remarkably, co‐treatment with these *Nocardioides* or *Streptomyces* strains alongside phage cocktails increased pathogen suppression by 40%–55% compared to phage‐only treatment, confirming the synergistic effect of these combined treatments (Wang et al. 2024). Further supporting these findings, additional studies showed that healthy tomato plants are associated with rhizosphere phage communities that contribute to the regulation of *R. solanacearum* populations (Yang et al. 2023). In addition, phage treatments have also been shown to affect bacterial abundance and diversity in the phyllosphere of tomato plants (Morella et al. 2018). Collectively, these studies highlight the pivotal role of the plant microbiome in maintaining plant health (Bakker et al. 2020; Pratama et al. 2020; Compant et al. 2024; Roca et al. 2024).

Regulations and guidelines for using phages as biocontrol agents in agriculture are still in their early stages and differ across countries (Toussaint et al. 2024). However, the safe use of phages in agriculture should ideally meet several requirements, including a lytic lifestyle (i.e., temperate phages can serve as reservoirs for antibiotic resistance and toxin genes in natural environments), the absence of genetic transduction capabilities, high progeny production, and the ability to specifically and efficiently target pathogenic bacteria. Furthermore, phage genomes should be free of virulence and antimicrobial resistance genes (Matilla et al. 2014; Svircev et al. 2018; Strathdee et al. 2023; Villalpando‐Aguilar et al. 2023; Siyanbola et al. 2024; Toussaint et al. 2024; Warring et al. 2024). A major concern regarding phage therapy is the potential development of phage resistance in target bacteria (Smith et al. 2023). This issue can be addressed by using phage cocktails with complementary functionalities, such as phages with varying host ranges and targeting different bacterial receptors (Svircev et al. 2018; Siyanbola et al. 2024; Warring et al. 2024). Phage cocktails also broaden the host spectrum and extend the longevity of phage treatments (Wang et al. 2019; Villalpando‐Aguilar et al. 2023; Ke et al. 2024; Warring et al. 2024). A recent study in *Microbial Biotechnology* investigated the emergence of phage resistance in the phytopathogen *P. syringae* using both individual phages and phage cocktails (Rabiey et al. 2024). Killing curve assays revealed bacterial resistance emerging as early as 16 h, with fluctuations indicative of an evolutionary arms race between phages and bacteria (i.e., resistant bacterial populations being countered by the evolution of infective phages). These fluctuations decreased and eventually disappeared as the number of different phages in the cocktails increased, suggesting that more complex phage cocktails can prevent arms race dynamics. Subsequent killing curve assays revealed a progressive increase in bacterial resistance to wild‐type phages over time, likely driven by the accumulation of mutations. An experimental evolution experiment further explored this by repeatedly co‐incubating *P. syringae* with either single phages or phage cocktails for 48 h, followed by transfers to fresh broth across 10 sequential cycles, with bacteria and phages recovered at each stage (Rabiey et al. 2024). The findings showed that bacteria from later transfers were more resistant to earlier phages (i.e., from previous transfers) than to contemporary (i.e., from present transfers) or subsequently evolved phages. Although bacterial resistance increased throughout the evolution experiment, it never reached 100%. Remarkably, wild‐type phages retained the ability to infect and kill co‐evolved *P. syringae* collected at different time points during the evolution experiment. The fitness costs of developing phage resistance were also investigated, revealing reduced growth rates and impaired plant virulence in a subset of resistant mutants. Genome sequencing of resistant isolates identified deletions, insertions, and mutations primarily in genes involved in lipopolysaccharide (LPS) synthesis, which was subsequently confirmed to serve as phage receptors through targeted gene deletions. Finally, since phages from later stages of the evolution experiment were able to infect wild‐type *P. syringae* , a phage cocktail composed of wild‐type and evolved phages was designed, which efficiently reduced the occurrence of bacterial resistance compared to the original formulation (Rabiey et al. 2024). Overall, the study by Rabiey et al. (2024) emphasises the importance of understanding bacterial resistance mechanisms for the development of effective phage‐based biocontrol strategies for sustainable agriculture practices. Remarkably, since mutations in phage receptors, such as LPS, have been shown to impact bacterial growth and phytopathogenicity (Warring et al. 2022; Rabiey et al. 2024), phage resistance may still offer benefits in managing plant diseases. Bacteria can also escape phage infection by entering a dormant persister state (Fernández‐García et al. 2024; Sanchez‐Torres et al. 2024), a factor that should also be considered when developing phage therapy strategies for agriculture. In this context, it has been suggested that combining phage therapy with anti‐persister compounds (e.g., mitomycin C, cisplatin, 5‐nitro‐3‐phenyl‐1H‐indol‐2‐yl‐methylamine hydrochloride) could significantly enhance the efficacy of treatments against bacterial infections (Sanchez‐Torres et al. 2024). Additionally, phages were shown to act synergistically with different anti‐microbials to combat antibiotic resistance (Warring et al. 2024).

The scientific community is increasingly emphasising microorganism‐based technologies, including phages, as key tools in plant pathogen biocontrol strategies to achieve the global goals outlined in the United Nations' 2030 Agenda for Sustainable Development (Crowther et al. 2024). This aspect becomes even more relevant in the current context of climate change, which is expected to intensify crop diseases due to factors such as the accelerated evolution and spread of phytopathogens and their vectors, as well as shifts in phytopathogen‐host interactions (Singh et al. 2023). In this regard, phage therapy is a very promising alternative to traditional methods for plant disease management (Figure 1), as phages have demonstrated effectiveness in biocontrol against phytopathogenic bacteria that impact numerous globally important crops (Holtappels et al. 2021; Strathdee et al. 2023; Siyanbola et al. 2024; Toussaint et al. 2024; Van Goethem et al. 2024; Warring et al. 2024). Furthermore, phages can act synergistically with other treatments (e.g., bacterial biocontrol agents, antimicrobials, endolysins, hygienic practices) to improve their performance in agricultural applications (Wang et al. 2017, 2024; Sieiro et al. 2020; Vu and Oh 2020; Warring et al. 2024). Notably, synthetic phages created through genome engineering were recognised as one of the top 10 emerging technologies of 2023 by the World Economic Forum and have proven effectiveness in biocontrol against phytopathogenic bacteria (Peng et al. 2024). However, despite the significant biotechnological potential of phage‐based approaches, limitations such as inconsistent field performance, stability issues, cost‐effective production challenges, the emergence of bacterial resistance as well as regulatory hurdles for their use and commercialization represent current obstacles that continue to impede their definitive adoption in agriculture applications. In this context, the Joint Research Centre has recently prepared a policy report for the European Parliament, providing the foundation for discussions on the use of phage therapy and biocontrol strategies (Toussaint et al. 2024). Remarkably, the first phage‐based biopesticide was approved in 2005 (Toussaint et al. 2024), and current projections suggest that the global biocontrol market will grow at an annual rate exceeding 16% (Biocontrol Global Market Report 2023). The regulation of phage‐based products varies significantly across countries worldwide, but the European Union has initiated a coordinated effort to harmonise the diverse national regulatory frameworks (Toussaint et al. 2024).

**FIGURE 1:**
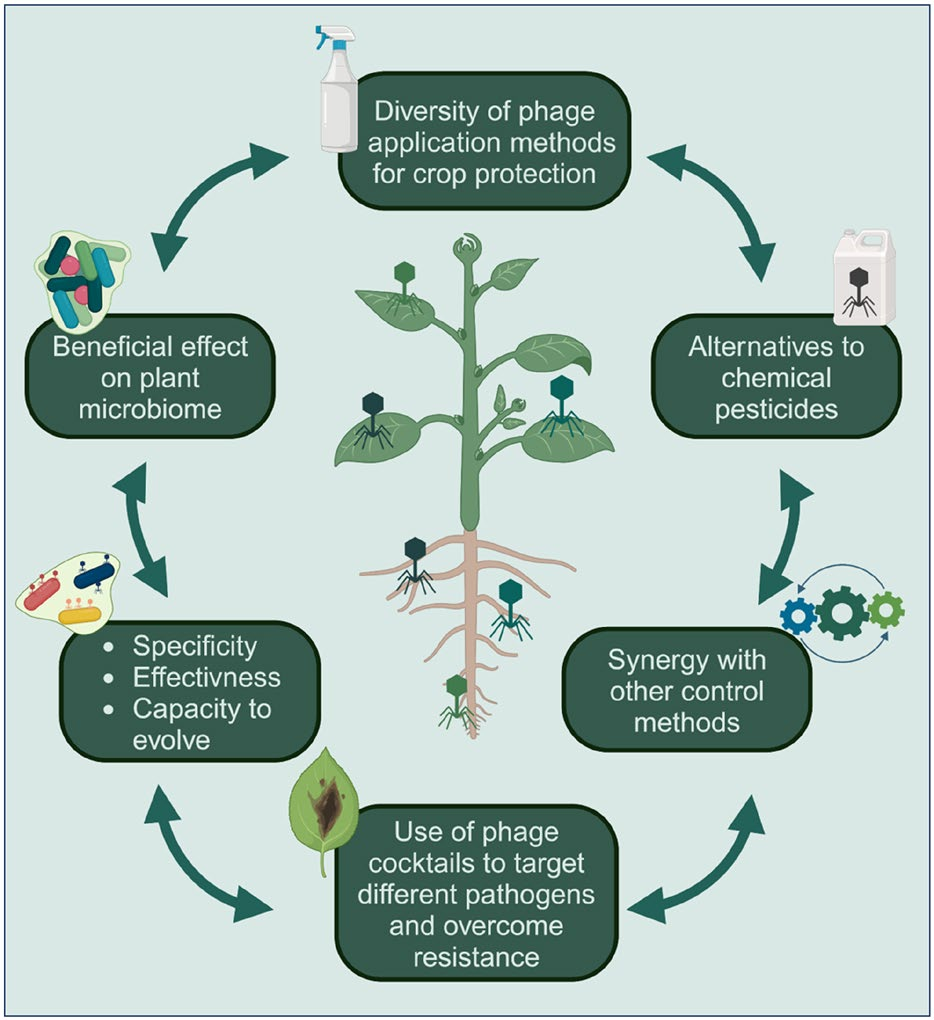
Advantages of phage therapy as a sustainable alternative in agriculture. Key benefits include bacterial specificity, effectiveness in reducing target phytopathogenic bacterial populations, and ability to co‐evolve with phage‐resistant bacteria. Phage therapy provides an eco‐friendly alternative to chemical pesticides, contributes positively to the plant microbiome and synergizes with other pest control methods. Phage cocktails can target different plant pathogens to improve biocontrol efficacy and help overcome resistance challenges. Created in BioRender by Czajkowski, R. (2025) https://BioRender.com/j76x743.

## Author Contributions

**Robert Czajkowski:** conceptualization, investigation, funding acquisition, writing – review and editing, project administration, resources. **Amalia Roca:** conceptualization, investigation, funding acquisition, writing – review and editing, project administration, resources. **Miguel A. Matilla:** conceptualization, investigation, funding acquisition, writing – review and editing, project administration, resources, writing – original draft.

## Conflicts of Interest

The authors declare no conflicts of interest.

## Data Availability

The authors have nothing to report.
